# Supplementary material for: Evolutionary pathways to SARS-CoV-2 resistance are opened and closed by epistasis acting on ACE2
Source: PLoS Biol. 2021 Dec 21;19(12):e3001510. doi: 10.1371/journal.pbio.3001510 (PMC8730403; doi:10.1371/journal.pbio.3001510)
Supplement: S1 Fig — ACE2 hydrolysis activity was measured using a fluorogenic peptide substrate (Mca-YVADAPK(Dnp)-OH) incubated with lysates of HEK293T cells for 2 hours. Cells were either untransfected or were transfected with a human ACE2 construct. Incubation of lysate–peptide mixture with a ACE2-specific inhibitor (DX600) reduced fluorescence attributable to ACE2 hydrolysis activity. N = 5 to 6 biological replicates. Standard error is shown. All data are available in S1 Data. ACE2, angiotensin converting enzyme 2. (DOCX) [file pbio.3001510.s001.docx]

Supplementary Figure S1.

**
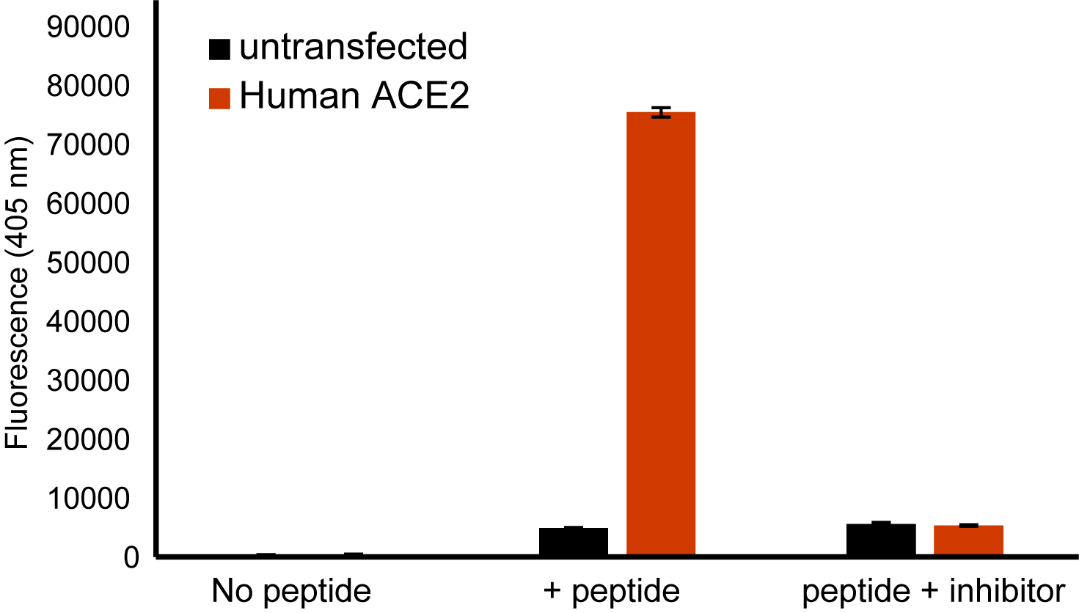
**

**Recombinant ACE2 hydrolysis activity in solubilized transfected HEK293T cells.** ACE2 hydrolysis activity was measured using a fluorogenic peptide substrate (Mca-YVADAPK(Dnp)-OH) incubated with lysates of HEK293T cells for 2 hours. Cells were either untransfected or were transfected with a human ACE2 construct. Incubation of lysate-peptide mixture with a ACE2-specific inhibitor (DX600) reduced fluorescence attributable to ACE2 hydrolysis activity. N=5-6 biological replicates. Standard error is shown. All data is available in S1 Data.
