# Supplementary material for: Evolutionary pathways to SARS-CoV-2 resistance are opened and closed by epistasis acting on ACE2
Source: PLoS Biol. 2021 Dec 21;19(12):e3001510. doi: 10.1371/journal.pbio.3001510 (PMC8730403; doi:10.1371/journal.pbio.3001510)

Supplementary Figure S2.

Conservation of angiotensin peptide sequences across mammalian species investigated in this study. Renin produces Angiotensin 1 by cleaving Angiotensinogen (*AGT* gene). Angiotensin 1 is subsequently cleaved by ACE, followed by ACE2.


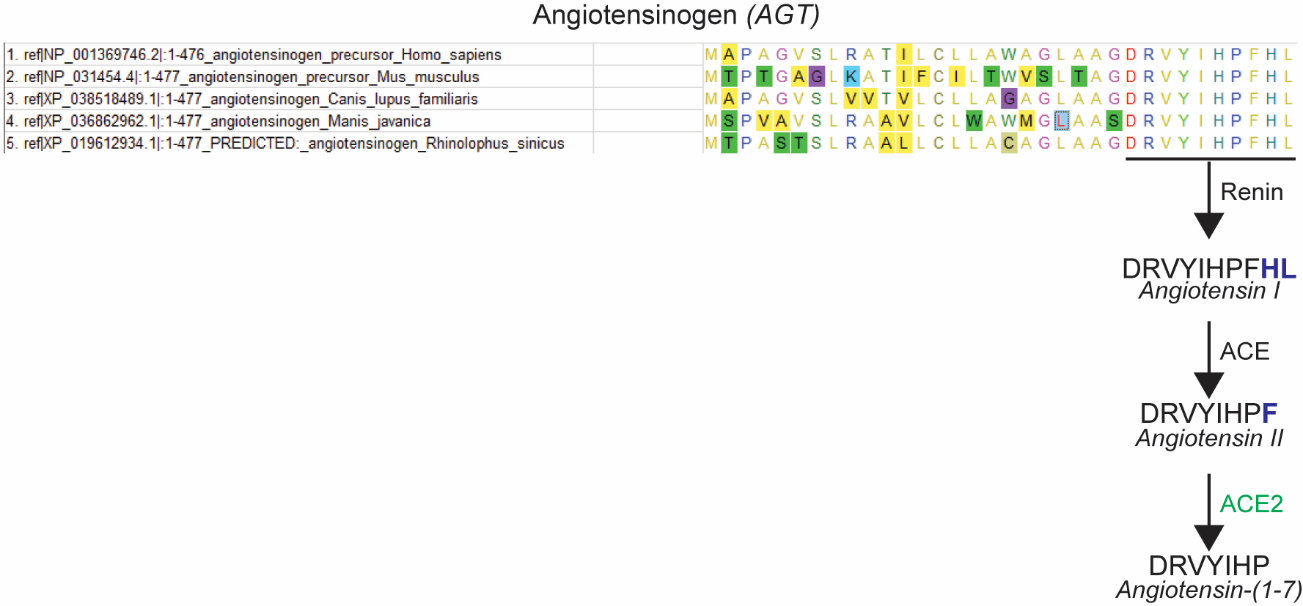

Supplement: S2 Fig — Renin produces angiotensin 1 by cleaving Angiotensinogen (AGT gene). Angiotensin 1 is subsequently cleaved by ACE, followed by ACE2. ACE2, angiotensin converting enzyme 2. (DOCX) [file pbio.3001510.s002.docx]
