# Supplementary material for: Evolutionary pathways to SARS-CoV-2 resistance are opened and closed by epistasis acting on ACE2
Source: PLoS Biol. 2021 Dec 21;19(12):e3001510. doi: 10.1371/journal.pbio.3001510 (PMC8730403; doi:10.1371/journal.pbio.3001510)

Supplementary Figure S3

Expression of ACE2-gfp orthologs in transfected HEK293T cells was assessed by flow cytometry (% of GFP-positive cells). These expressed ACE2 proteins were used in hydrolysis assays shown in figure 2 (**A**) and figure 4 (**B**). Human ACE2 served as an internal control in each separate assay.


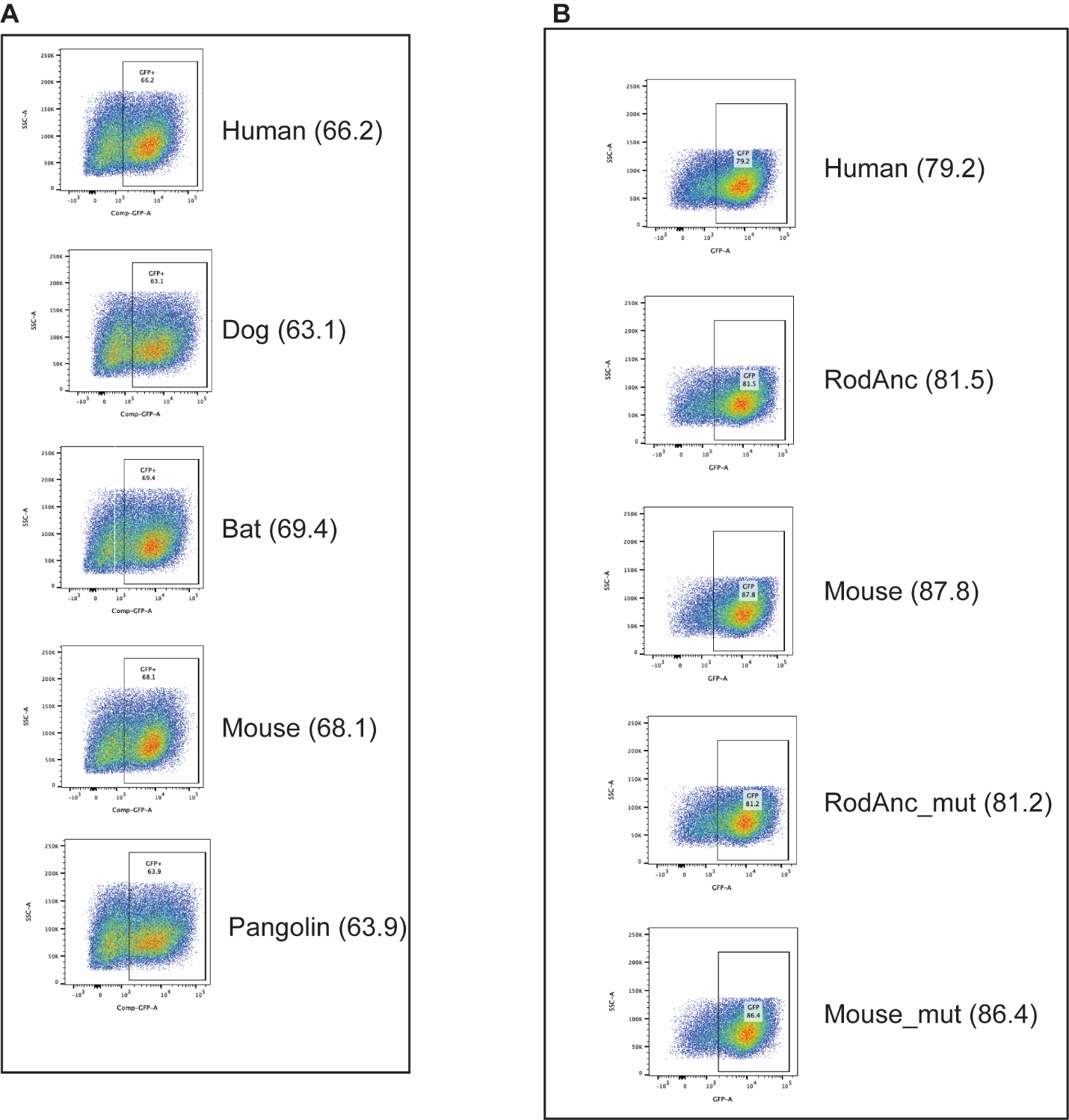

Supplement: S3 Fig — These expressed ACE2 proteins were used in hydrolysis assays. Human ACE2 served as an internal control in each separate assay. ACE2, angiotensin converting enzyme 2. (DOCX) [file pbio.3001510.s003.docx]
