# Supplementary material for: Evolutionary pathways to SARS-CoV-2 resistance are opened and closed by epistasis acting on ACE2
Source: PLoS Biol. 2021 Dec 21;19(12):e3001510. doi: 10.1371/journal.pbio.3001510 (PMC8730403; doi:10.1371/journal.pbio.3001510)

Supplementary Figure S4.

Maximum likelihood phylogeny used in PAML analyses. aLRT-SH like branch support values (PhyML) are shown. Note that a basal trichotomy was artificially induced to accommodate input file requirements. All data is available in S1 Data.


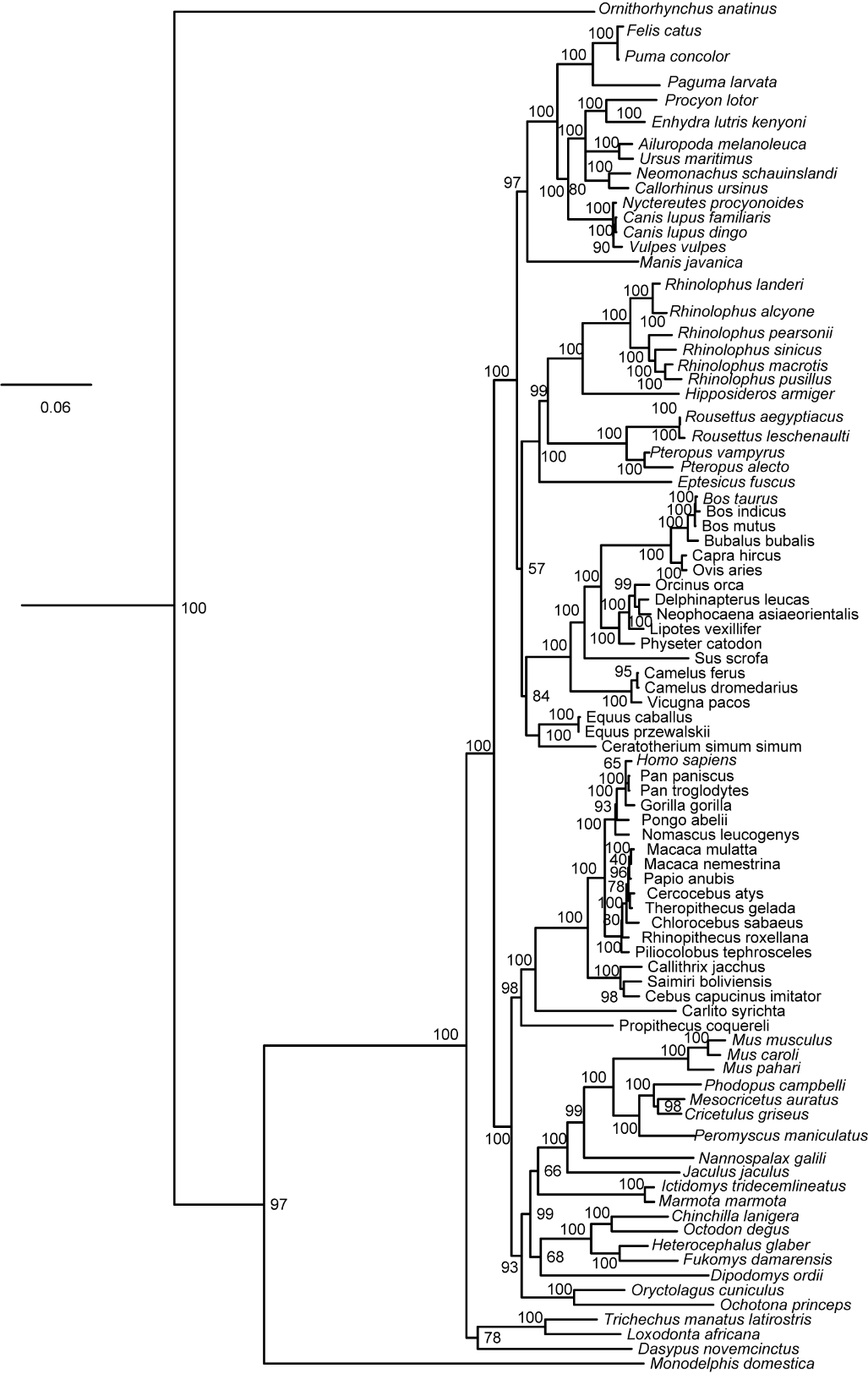

Supplement: S4 Fig — aLRT-SH like branch support values (IQ-Tree) are shown. Note that a basal trichotomy was artificially induced to accommodate input file requirements. All data are available in S1 Data. (DOCX) [file pbio.3001510.s004.docx]
