# Supplementary material for: Evolutionary pathways to SARS-CoV-2 resistance are opened and closed by epistasis acting on ACE2
Source: PLoS Biol. 2021 Dec 21;19(12):e3001510. doi: 10.1371/journal.pbio.3001510 (PMC8730403; doi:10.1371/journal.pbio.3001510)
Supplement: S5 Fig — Western blots of immunopreciptations and cell lysates of HEK293T cells co-transfected with an Fc-tagged SARS-CoV-2 S protein RBD and 1D4-tagged (C9) human ACE2 construct. ACE2, angiotensin converting enzyme 2; RBD, receptor-binding domain; SARS-CoV-1, Severe Acute Respiratory Syndrome Coronavirus; SARS-CoV-2, Severe Acute Respiratory Syndrome Coronavirus 2. (DOCX) [file pbio.3001510.s005.docx]

Supplementary Figure S5.

**
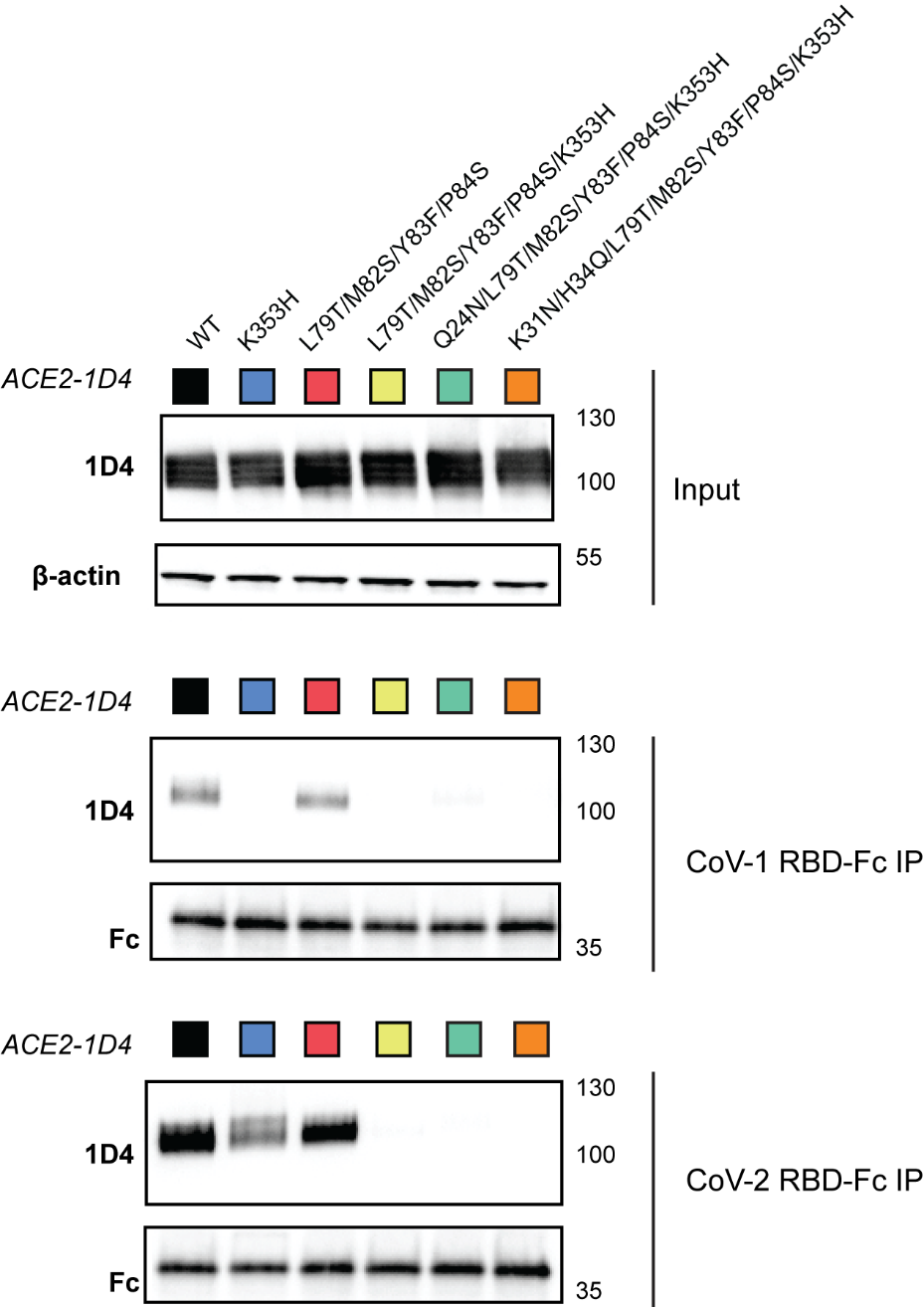
**

**Targeted mutations to human ACE2 disrupt binding to the RBD of SARS-CoV-1 and SARS-CoV-2.** Western blots of immunopreciptations and cell lysates of HEK293T cells co-transfected with an Fc-tagged SARS-CoV-2 S protein RBD, and 1D4-tagged (C9) human ACE2 construct.
