# Supplementary material for: Evolutionary pathways to SARS-CoV-2 resistance are opened and closed by epistasis acting on ACE2
Source: PLoS Biol. 2021 Dec 21;19(12):e3001510. doi: 10.1371/journal.pbio.3001510 (PMC8730403; doi:10.1371/journal.pbio.3001510)

Supplementary Figure S6.

Maximum likelihood phylogeny used in ancestral reconstruction of Rodent ACE2. aLRT-SH like branch support values (IQ-Tree) are shown. All data is available in S1 Data.


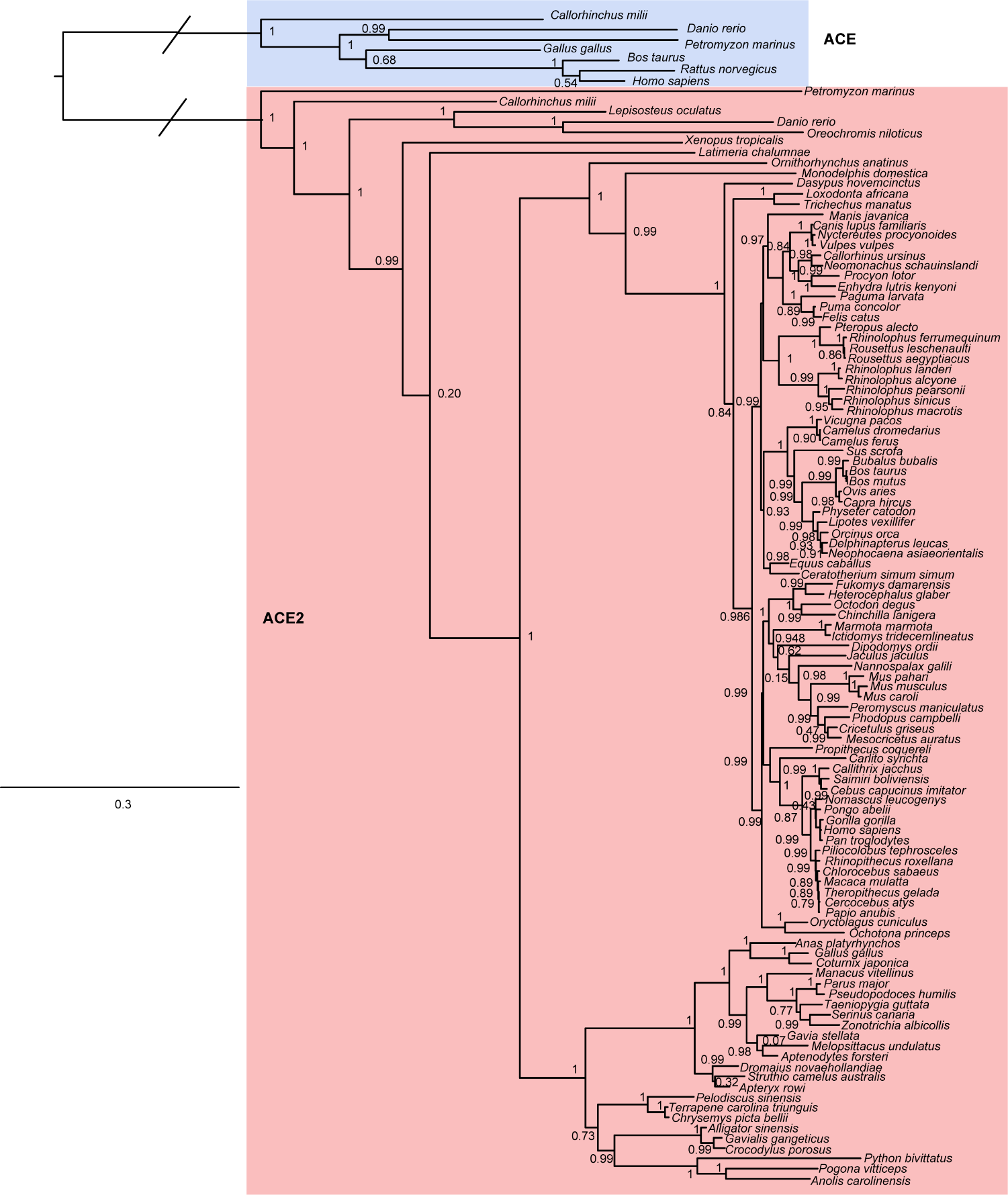

Supplement: S6 Fig — aLRT-SH like branch support values (PhyML) are shown. All data are available in S1 Data. ACE2, angiotensin converting enzyme 2. (DOCX) [file pbio.3001510.s006.docx]
