# Supplementary material for: Evolutionary pathways to SARS-CoV-2 resistance are opened and closed by epistasis acting on ACE2
Source: PLoS Biol. 2021 Dec 21;19(12):e3001510. doi: 10.1371/journal.pbio.3001510 (PMC8730403; doi:10.1371/journal.pbio.3001510)

Supplementary Figure S7.

Species phylogeny and least squares linear regression using phylogenetically independent contrasts of systolic blood pressure and body mass. All data is available in S1 Data.


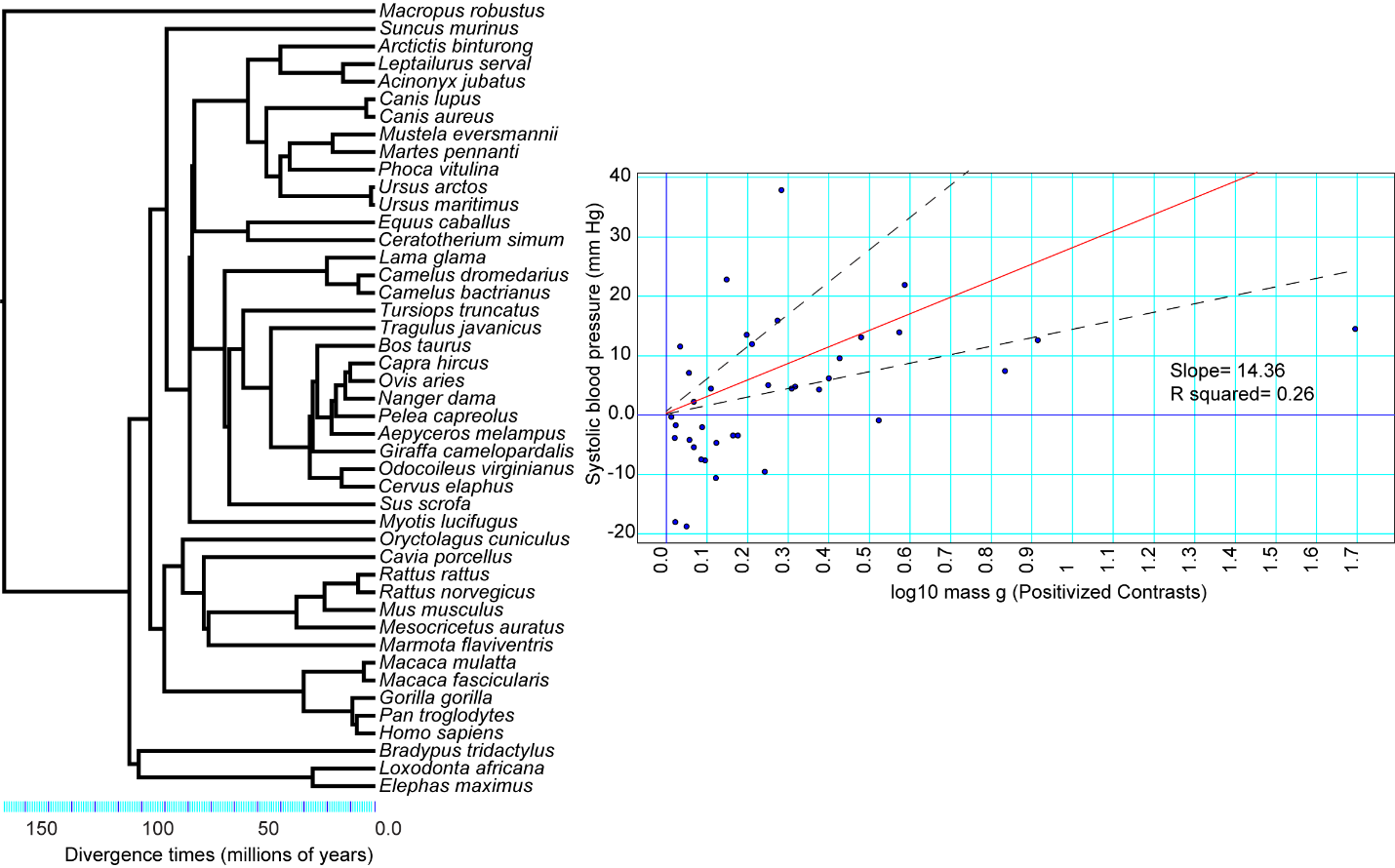

Supplement: S7 Fig — All data are available in S1 Data. (DOCX) [file pbio.3001510.s007.docx]
