# Supplementary material for: Evolutionary pathways to SARS-CoV-2 resistance are opened and closed by epistasis acting on ACE2
Source: PLoS Biol. 2021 Dec 21;19(12):e3001510. doi: 10.1371/journal.pbio.3001510 (PMC8730403; doi:10.1371/journal.pbio.3001510)
Supplement: S1 Table — ACE2, angiotensin converting enzyme 2. (DOCX) [file pbio.3001510.s008.docx]

Supplementary Table 1. ACE2 accession numbers used in dN/dS estimates

| scientific name | accession |
| --- | --- |
| *Ornithorhynchus_anatinus* | XM_001515547.4 |
| *Monodelphis domestica* | XM_007500874.2 |
| *Dasypus novemcinctus* | XM_004449067.3 |
| *Loxodonta africana* | XM_023555192.1 |
| *Trichechus manatus latirostris* | XM 004386324.2 |
| *Manis javanica* | XM 017650263.1 |
| *Paguma larvata* | AY881174.1 |
| *Felis catus* | AB211997.1 |
| *Puma concolor* | XM 025934632.1 |
| *Procyon lotor* | AB211998 |
| *Enhydra lutris kenyoni* | XM 022518370.1 |
| *Ailuropoda melanoleuca* | XM 002930611.3 |
| *Ursus maritimus* | XM 008696415.1 |
| *Neomonachus schauinslandi* | XM 021680805.1 |
| *Callorhinus ursinus* | XM 025857612.1 |
| *Nyctereutes procyonoides* | EU024940.1 |
| *Vulpes vulpes* | XM 025986727.1 |
| *Canis lupus familiaris* | XM 014111329.2 |
| *Canis lupus dingo* | XM 025437140.1 |
| *Eptesicus fuscus* | XM 008154928.2 |
| *Hipposideros armiger* | XM 019667391.1 |
| *Rhinolophus landeri* | KR559015.1 |
| *Rhinolophus alcyone* | KR559016.1 |
| *Rhinolophus pearsonii* | EF569964.1 |
| *Rhinolophus sinicus* | GQ262791.1 |
| *Rhinolophus macrotis* | GQ999932.1 |
| *Rhinolophus pusillus* | GQ999938.1 |
| *Rhinolophus ferrumequinum* | FJ598617.1 |
| *Rousettus leschenaulti* | AB299376.1 |
| *Rousettus aegyptiacus* | XM 016118926.1 |
| *Pteropus vampyrus* | XM 011362973.2 |
| *Pteropus alecto* | XM 006911647.1 |
| *Sus scrofa* | GQ262781.1 |
| *Bubalus bubalis* | XM 006041540.2 |
| *Bos mutus* | XM 005903111.1 |
| *Bos taurus* | BC105340.1 |
| *Capra hircus* | XM 005701072.3 |
| *Ovis aries* | XM 012106267.3 |
| *Physeter catodon* | XM 024115511.2 |
| *Lipotes vexillifer* | XM 007466327.1 |
| *Orcinus orca* | XM 004269657.1 |
| *Delphinapterus leucas* | XM 022562652.2 |
| *Neophocaena asiaeorientalis* | XM 024744126.1 |
| *Vicugna pacos* | XM 006212647.3 |
| *Camelus ferus* | XM 006194201.2 |
| *Camelus dromedarius* | XM 010993415.2 |
| *Ceratotherium simum simum* | XM 004435149.2 |
| *Equus caballus* | XM 001490191.5 |
| *Equus przewalskii* | XM 008544773.1 |
| *Propithecus coquereli* | XM 012638732.1 |
| *Carlito syrichta* | XM 008064619.1 |
| *Callithrix jacchus* | XM 017968359.1 |
| *Saimiri boliviensis* | XM 010336623.1 |
| *Cebus capucinus imitator* | XM 017512376.1 |
| *Nomascus leucogenys* | XM 003261084.3 |
| *Pongo abelii* | NM 001131132.2 |
| *Gorilla gorilla gorilla* | XM 019019204.1 |
| *Homo sapiens* | NM 001371415.1 |
| *Pan paniscus* | XM 008974180.1 |
| *Pan troglodytes* | XM 016942979.1 |
| *Piliocolobus tephrosceles* | XM 023199053.2 |
| *Rhinopithecus roxellana* | XM 010366065.2 |
| *Chlorocebus sabaeus* | XM 007991113.1 |
| *Papio anubis* | XM 021933040.1 |
| *Macaca mulatta* | XM 015126958.2 |
| *Macaca nemestrina* | XM 011735203.2 |
| *Cercocebus atys* | XM 012035808.1 |
| *Theropithecus gelada* | XM 025372062.1 |
| *Oryctolagus cuniculus* | GQ262787.1 |
| *Ochotona princeps* | XM 004597492.2 |
| *Dipodomys ordii* | XM 013032118.1 |
| *Heterocephalus glaber* | XM 004866100 |
| *Fukomys damarensis* | XM 010645175.2 |
| *Chinchilla lanigera* | NM 001282361.1 |
| *Octodon degus* | XM 023719547.1 |
| *Ictidomys tridecemlineatus* | XM 005315994.3 |
| *Marmota marmota* | XM 015488054.1 |
| *Jaculus jaculus* | XM 004671466.2 |
| *Nannospalax galili* | XM 008840876.2 |
| *Phodopus campbelli* | GQ262790.1 |
| *Mesocricetus auratus* | GQ262794.1 |
| *Cricetulus griseus* | XM 003503235.4 |
| *Peromyscus maniculatus bairdii* | XM 006973207.2 |
| *Mus caroli* | XM 021153479.2 |
| *Mus pahari* | XM 021188276.2 |
| *Mus musculus* | NM 001130513.1 |
