# Supplementary material for: Evolutionary pathways to SARS-CoV-2 resistance are opened and closed by epistasis acting on ACE2
Source: PLoS Biol. 2021 Dec 21;19(12):e3001510. doi: 10.1371/journal.pbio.3001510 (PMC8730403; doi:10.1371/journal.pbio.3001510)
Supplement: S2 Table — ACE2, angiotensin converting enzyme 2. (DOCX) [file pbio.3001510.s009.docx]

Supplementary Table 2.

Analyses of selection on Mammalian *ACE2* using PAML random sites models.

| **Model** | **ΔAIC^1^** | ***ln*L** | **Parameters^2^** | | | **Null** | ***p*** **[df]^3^** |
| --- | --- | --- | --- | --- | --- | --- | --- |
|  |  |  | ***ω*_0_/p** | ***ω*_1_/q** | ***ω*_2_/*ω*_p_** |  |  |
| M0 | 3746.8 | -36519.69 | 0.31 | - | - | N/A | - |
| M1a | 569.2 | -34929.93 | 0.08 (67%) | 1.00 (33%) | - | M0 | **0.000** [1] |
| M2a | 369.6 | -34828.09 | 0.08 (66%) | 1.00 (29%) | 2.39 (5%) | M1a | **0.000** [2] |
| M3 | 110.3 | -34697.44 | 0.04 (57%) | 0.48 (31%) | 1.51 (12%) | M2 | **0.000** [1] |
| M7 | 186.8 | -34738.71 | 0.24 | 0.55 | - | N/A | - |
| M8a | 141.3 | -34714.97 | 0.35 | 1.66 | 1.00 | N/A | - |
| **M8** | **0.0*** | -34643.31 | 0.29 | 0.87 | 1.84 | M7 | **0.000** [2] |
|  |  |  |  |  |  | M8a | **0.000** [1] |

^1^All ΔAIC values are calculated from the lowest AIC model. The best fits are bolded with an asterisk (*).

^2^*ω* values of each site class are shown are shown for model M0-M3 (*ω*_0_– *ω*_2_) with the proportion of each site class in parentheses. For M7 and M8, the shape parameters, p and q, which describe the beta distribution are listed instead. In addition, the *ω* value for the positively selected site class (*ω*_p_, with the proportion of sites in parentheses) is shown for M8.

^3^Significant *p*-values (α ≤ 0.05) are bolded. Degrees of freedom are given in square brackets after the *p*-values. Significance was determined through a likelihood-ratio test of null and alternative models, with reference to a χ^2^ distribution.

Abbreviations—***ln*L**, ln Likelihood; ***p***, *p-*value; **N/A**, not applicable.
