# Supplementary material for: Evolutionary pathways to SARS-CoV-2 resistance are opened and closed by epistasis acting on ACE2
Source: PLoS Biol. 2021 Dec 21;19(12):e3001510. doi: 10.1371/journal.pbio.3001510 (PMC8730403; doi:10.1371/journal.pbio.3001510)

Supplementary Table 3.

Results of BUSTED (HyPhy) analyses of Mammalian *ACE2*. This model accounts for synonymous rate variation (SRV). *Log* L values demonstrate that the unconstrained model performs better than the constrained, specifically due to the inclusion of a positive selection omega site category (ω_3_).


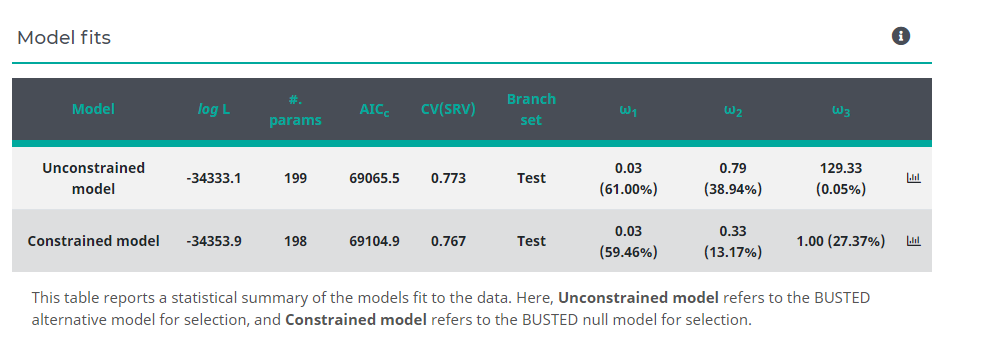

Supplement: S3 Table — This model accounts for synonymous rate variation (SRV). Log L values demonstrate that the unconstrained model performs better than the constrained, specifically due to the inclusion of a positive selection omega site category (ω3). ACE2, angiotensin converting enzyme 2. (DOCX) [file pbio.3001510.s010.docx]
