# Supplementary material for: Evolutionary pathways to SARS-CoV-2 resistance are opened and closed by epistasis acting on ACE2
Source: PLoS Biol. 2021 Dec 21;19(12):e3001510. doi: 10.1371/journal.pbio.3001510 (PMC8730403; doi:10.1371/journal.pbio.3001510)
Supplement: S4 Table — ACE2, angiotensin converting enzyme 2. (DOCX) [file pbio.3001510.s011.docx]

Supplementary Table 4.

Analyses of selection on Mammalian *ACE2* without bat (Chioptera) sequences using PAML random sites models.

| **Model** | **ΔAIC^1^** | ***ln*L** | **Parameters^2^** | | | **Null** | ***p*** **[df]^3^** |
| --- | --- | --- | --- | --- | --- | --- | --- |
|  |  |  | ***ω*_0_/p** | ***ω*_1_/q** | ***ω*_2_/*ω*_p_** |  |  |
| M0 | 2958.8 | -30888.13 | 0.28 | - | - | N/A | - |
| M1a | 426.8 | -29621.11 | 0.08 (72%) | 1.00 (27%) | - | M0 | **0.000** [1] |
| M2a | 332.7 | -29572.08 | 0.08 (71%) | 1.00 (25%) | 2.27 (4%) | M1a | **0.000** [2] |
| M3 | 71.1 | -29440.28 | 0.02 (53%) | 0.34 (32%) | 1.22 (15%) | M2 | **0.000** [1] |
| M7 | 114.2 | -29464.83 | 0.23 | 0.60 | - | N/A | - |
| M8a | 72.9 | -29443.16 | 0.36 | 2.15 | 1.00 | N/A | - |
| **M8** | **0.0*** | -29405.73 | 0.28 | 0.99 | 1.72 | M7 | **0.000** [2] |
|  |  |  |  |  |  | M8a | **0.000** [1] |

^1^All ΔAIC values are calculated from the lowest AIC model. The best fits are bolded with an asterisk (*).

^2^*ω* values of each site class are shown are shown for model M0-M3 (*ω*_0_– *ω*_2_) with the proportion of each site class in parentheses. For M7 and M8, the shape parameters, p and q, which describe the beta distribution are listed instead. In addition, the *ω* value for the positively selected site class (*ω*_p_, with the proportion of sites in parentheses) is shown for M8.

^3^Significant *p*-values (α ≤ 0.05) are bolded. Degrees of freedom are given in square brackets after the *p*-values. Significance was determined through a likelihood-ratio test of null and alternative models, with reference to a χ^2^ distribution.

Abbreviations—***ln*L**, ln Likelihood; ***p***, *p-*value; **N/A**, not applicable.
