# Supplementary material for: Evolutionary pathways to SARS-CoV-2 resistance are opened and closed by epistasis acting on ACE2
Source: PLoS Biol. 2021 Dec 21;19(12):e3001510. doi: 10.1371/journal.pbio.3001510 (PMC8730403; doi:10.1371/journal.pbio.3001510)
Supplement: S5 Table — ACE2, angiotensin converting enzyme 2. (DOCX) [file pbio.3001510.s012.docx]

Supplementary Table 5.

Positively selected sites in mammalian ACE2

|  | M8_Mammals^1^ | | M8_Mammals_no bats^2^ | |
| --- | --- | --- | --- | --- |
|  | ***ω*_p_^3^** | **pos probs^4^** | ***ω*_p_** | **pos probs** |
| 24 | 2.085 | 0.99976 | 1.491 | 0.98529 |
| 79 | 1.724 | 0.75524 | 1.432 | 0.89081 |
| 82 | 1.21 | 0.40096 | 1.121 | 0.44223 |
| 83 | 0.394 | 0.00005 | 0.334 | 0.0001 |
| 84 | 1.397 | 0.5264 | 1.147 | 0.4914 |
| 353 | 0.193 | 0 | 0.235 | 0 |

^1^Refers to M8 analysis of all mammalian ACE2 sequences (Table S2)

^2^Refers to M8 analysis the mammalian ACE2 dataset, but without bat sequences (Table S5)

^3^*ω* values (dN/dS) for the positively selected site class (*ω*_p_) is shown for each respective M8 analysis.

^4^Posterior probabilities from Bayes Empirical Bayes analysis supporting each ω_p_ category assignment
