# Supplementary material for: Evolutionary pathways to SARS-CoV-2 resistance are opened and closed by epistasis acting on ACE2
Source: PLoS Biol. 2021 Dec 21;19(12):e3001510. doi: 10.1371/journal.pbio.3001510 (PMC8730403; doi:10.1371/journal.pbio.3001510)
Supplement: S6 Table — ACE2, angiotensin converting enzyme 2; CmD, clade model D. (DOCX) [file pbio.3001510.s013.docx]

Supplementary Table 6.

Results of Clade Model D (CmD) analyses of Mammalian *ACE2* under various partitions.

| **Model &**  **Foreground^1^** | **ΔAIC^2^** | ***ln*L** | **Parameters^3^** | | | **Null** | ***p*** **[df]^4^** |
| --- | --- | --- | --- | --- | --- | --- | --- |
|  |  |  | ***ω*_0_** | ***ω*_1_** | ***ω*_2_/*ω*_d_** |  |  |
| M3 | 6.51 | -722.34 | 0.00 (0%) | 0.14 (25%) | 1.17 (75%) | N/A | - |
| CmD_*Bats* | 4.31 | -720.24 | 0.00 (0%) | 0.14 (25%) | 0.97 (75%)  Bats: 2.44 | M3 | **0.04** [1] |
| CmD_*Rodents* | 1.66 | -718.92 | 0.00 (0%) | 0.14 (25%) | 1.63 (75%)  Rodents: 0.52 | M3 | **0.000** [1] |
| CmD_*Primates* | 5.63 | -720.90 | 0.00 (0%) | 0.14 (25%) | 1.31 (75%)  Primates: 0.39 | M3 | 0.09 [1] |
| CmD_*RodentsBats* | 1.91 | -718.04 | 0.00 (0%) | 0.13 (25%) | 1.37 (75%)  Rodents: 0.52 Bats: 2.44 | M3 | **0.03** [2] |
| CmD_*RodentsBatsPrimates* | **0.00*** | -716.07 | 0.00 (0%) | 0.13 (25%) | 1.77 (75%)  Rodents: 0.52  Bats: 2.44  Primates: 0.42 | M3 | **0.006** [3] |

^1^The foreground partition is listed after the underscore for the clade models and consists of either: the clade of ‘Bats, ‘Rodents’, or the clade ‘Primates’. In any partitioning scheme, the entire clade was tested, and all non-foreground data are present in the background partition.

^2^All ΔAIC values are calculated from the lowest AIC model. The best fits are bolded with an asterisk (*).

^3^*ω*_d_ is the divergent site class, which has a separate value for the foreground and background partitions.

^4^Significant *p*-values (α ≤ 0.05) are bolded. Degrees of freedom are given in square brackets after the *p*-values. Significance was determined through a likelihood-ratio test of null and alternative models, with reference to a χ^2^ distribution.

Abbreviations—***ln*L**, ln Likelihood; ***p***, *p-*value; **AIC**, Akaike information criterion
