# Supplementary material for: Evolutionary pathways to SARS-CoV-2 resistance are opened and closed by epistasis acting on ACE2
Source: PLoS Biol. 2021 Dec 21;19(12):e3001510. doi: 10.1371/journal.pbio.3001510 (PMC8730403; doi:10.1371/journal.pbio.3001510)
Supplement: S7 Table — ACE, angiotensin converting enzyme; ACE2, angiotensin converting enzyme 2. (DOCX) [file pbio.3001510.s014.docx]

Supplementary Table 7. ACE and ACE2 accession numbers used in ancestral reconstruction

| scientific name | accession | Gene |
| --- | --- | --- |
| *Petromyzon marinus* | XM 032951728.1 | ACE |
| *Callorhinchus milii* | XM 007907341.1 |  |
| *Danio rerio* | XM 689244.9 |  |
| *Gallus gallus* | NM 001167732.1 |  |
| *Bos taurus* | NM 001206668.1 |  |
| *Homo sapiens* | NM 000789.4 |  |
| *Rattus norvegicus* | NM 012544.1 |  |
| *Petromyzon marinus* | XM 032979141.1 | ACE2 |
| *Callorhinchus milii* | XM 007891654.1 |  |
| *Lepisosteus oculatus* | XM 006639122.2 |  |
| *Danio rerio* | NM 001007297.1 |  |
| *Latimeria chalumnae* | XM 005997853.2 |  |
| *Oreochromis niloticus* | XM 005466811.4 |  |
| *Xenopus tropicalis* | XM 002938247.4 |  |
| *Anolis carolinensis* | XM 008107249.1 |  |
| *Python bivittatus* | XM 007431880.3 |  |
| *Pogona vitticeps* | XM 020786763.1 |  |
| *Pelodiscus sinensis* | XM 006122829.3 |  |
| *Chrysemys picta bellii* | XM 005287788.2 |  |
| *Terrapene carolina triunguis* | XM 026648166.1 |  |
| *Alligator sinensis* | XM 025210843.1 |  |
| *Gavialis gangeticus* | XM 019525515.1 |  |
| *Crocodylus porosus* | XM 019529281.1 |  |
| *Struthio camelus australis* | XM 009669200.1 |  |
| *Dromaius novaehollandiae* | XM 026120784.1 |  |
| *Apteryx rowi* | XM 026087161.1 |  |
| *Coturnix japonica* | XM 015886577.2 |  |
| *Anas platyrhynchos* | XM 013094461.3 |  |
| *Gallus gallus* | MK560199.1 |  |
| *Taeniopygia guttata* | XM 002194267.4 |  |
| *Manacus vitellinus* | XM 018084005.2 |  |
| *Parus major* | XM 015631329.1 |  |
| *Serinus canaria* | XM 009089674.3 |  |
| *Aptenodytes forsteri* | XM 009276865.1 |  |
| *Gavia stellata* | XM 009817825.1 |  |
| *Melopsittacus undulatus* | XM 005151459.1 |  |
| *Zonotrichia albicollis* | XM 005491775.2 |  |
| *Pseudopodoces humilis* | XM 005516655.1 |  |
| *Ornithorhynchus_anatinus* | XM_001515547.4 |  |
| *Monodelphis domestica* | XM_007500874.2 |  |
| *Dasypus novemcinctus* | XM_004449067.3 |  |
| *Loxodonta africana* | XM_023555192.1 |  |
| *Trichechus manatus latirostris* | XM 004386324.2 |  |
| *Manis javanica* | XM 017650263.1 |  |
| *Felis catus* | AB211997.1 |  |
| *Puma concolor* | XM 025934632.1 |  |
| *Procyon lotor* | AB211998 |  |
| *Enhydra lutris kenyoni* | XM 022518370.1 |  |
| *Ursus maritimus* | XM 008696415.1 |  |
| *Neomonachus schauinslandi* | XM 021680805.1 |  |
| *Callorhinus ursinus* | XM 025857612.1 |  |
| *Nyctereutes procyonoides* | EU024940.1 |  |
| *Vulpes vulpes* | XM 025986727.1 |  |
| *Canis lupus familiaris* | XM 014111329.2 |  |
| *Eptesicus fuscus* | XM 008154928.2 |  |
| *Rhinolophus landeri* | KR559015.1 |  |
| *Rhinolophus alcyone* | KR559016.1 |  |
| *Rhinolophus pearsonii* | EF569964.1 |  |
| *Rhinolophus sinicus* | GQ262791.1 |  |
| *Rhinolophus macrotis* | GQ999932.1 |  |
| *Rhinolophus ferrumequinum* | FJ598617.1 |  |
| *Rousettus leschenaulti* | AB299376.1 |  |
| *Rousettus aegyptiacus* | XM 016118926.1 |  |
| *Pteropus alecto* | XM 006911647.1 |  |
| *Sus scrofa* | GQ262781.1 |  |
| *Bubalus bubalis* | XM 006041540.2 |  |
| *Bos mutus* | XM 005903111.1 |  |
| *Bos taurus* | BC105340.1 |  |
| *Capra hircus* | XM 005701072.3 |  |
| *Ovis aries* | XM 012106267.3 |  |
| *Physeter catodon* | XM 024115511.2 |  |
| *Lipotes vexillifer* | XM 007466327.1 |  |
| *Orcinus orca* | XM 004269657.1 |  |
| *Delphinapterus leucas* | XM 022562652.2 |  |
| *Neophocaena asiaeorientalis* | XM 024744126.1 |  |
| *Vicugna pacos* | XM 006212647.3 |  |
| *Camelus ferus* | XM 006194201.2 |  |
| *Camelus dromedarius* | XM 010993415.2 |  |
| *Ceratotherium simum simum* | XM 004435149.2 |  |
| *Equus caballus* | XM 001490191.5 |  |
| *Propithecus coquereli* | XM 012638732.1 |  |
| *Carlito syrichta* | XM 008064619.1 |  |
| *Callithrix jacchus* | XM 017968359.1 |  |
| *Saimiri boliviensis* | XM 010336623.1 |  |
| *Cebus capucinus imitator* | XM 017512376.1 |  |
| *Nomascus leucogenys* | XM 003261084.3 |  |
| *Pongo abelii* | NM 001131132.2 |  |
| *Gorilla gorilla gorilla* | XM 019019204.1 |  |
| *Homo sapiens* | NM 001371415.1 |  |
| *Pan troglodytes* | XM 016942979.1 |  |
| *Piliocolobus tephrosceles* | XM 023199053.2 |  |
| *Rhinopithecus roxellana* | XM 010366065.2 |  |
| *Chlorocebus sabaeus* | XM 007991113.1 |  |
| *Papio anubis* | XM 021933040.1 |  |
| *Macaca mulatta* | XM 015126958.2 |  |
| *Macaca nemestrina* | XM 011735203.2 |  |
| *Cercocebus atys* | XM 012035808.1 |  |
| *Theropithecus gelada* | XM 025372062.1 |  |
| *Oryctolagus cuniculus* | GQ262787.1 |  |
| *Ochotona princeps* | XM 004597492.2 |  |
| *Dipodomys ordii* | XM 013032118.1 |  |
| *Heterocephalus glaber* | XM 004866100 |  |
| *Fukomys damarensis* | XM 010645175.2 |  |
| *Chinchilla lanigera* | NM 001282361.1 |  |
| *Octodon degus* | XM 023719547.1 |  |
| *Ictidomys tridecemlineatus* | XM 005315994.3 |  |
| *Marmota marmota* | XM 015488054.1 |  |
| *Jaculus jaculus* | XM 004671466.2 |  |
| *Nannospalax galili* | XM 008840876.2 |  |
| *Phodopus campbelli* | GQ262790.1 |  |
| *Mesocricetus auratus* | GQ262794.1 |  |
| *Cricetulus griseus* | XM 003503235.4 |  |
| *Peromyscus maniculatus bairdii* | XM 006973207.2 |  |
| *Mus caroli* | XM 021153479.2 |  |
| *Mus pahari* | XM 021188276.2 |  |
| *Mus musculus* | NM 001130513.1 |  |
