# Supplementary material for: Evolutionary pathways to SARS-CoV-2 resistance are opened and closed by epistasis acting on ACE2
Source: PLoS Biol. 2021 Dec 21;19(12):e3001510. doi: 10.1371/journal.pbio.3001510 (PMC8730403; doi:10.1371/journal.pbio.3001510)
Supplement: S8 Table — ACE2, angiotensin converting enzyme 2. (DOCX) [file pbio.3001510.s015.docx]

Supplementary Table 8.

Results of random sites analyses of vertebrate *ACE2*, with the best fitting mode (M8) used for the ancestral reconstruction of mammalian ACE2.

| **Model** | **ΔAIC** | ***ln*L** | **Parameters^2^** | | | **Null** | ***p*** **[df]^3^** |
| --- | --- | --- | --- | --- | --- | --- | --- |
|  |  |  | ***ω*_0_/p** | ***ω*_1_/q** | ***ω*_2_/*ω*_p_** |  |  |
| M3 | 231.6 | -63042.66 | 0.02 (42%) | 0.23 (33%) | 0.78 (25%) | N/A | - |
| M7 | 46.32 | -62953.03 | 0.35 | 0.84 | - | N/A | - |
| M8a | **53.04** | -62955.39 | 0.49 | 1.84 | 1 |  | **-** |
| **M8** | **0.0*** | -62927.87 | 0.41 | 1.35 | 1.22 | M7 | **0.000** [2] |
|  |  |  |  |  |  | M8a | **0.000** [1] |

^1^All ΔAIC values are calculated from the lowest AIC model. The best fits are bolded with an asterisk (*).

^2^*ω* values of each site class are shown are shown for model M0-M3 (*ω*_0_– *ω*_2_) with the proportion of each site class in parentheses. For M7 and M8, the shape parameters, p and q, which describe the beta distribution are listed instead. In addition, the *ω* value for the positively selected site class (*ω*_p_, with the proportion of sites in parentheses) is shown for M8.

^3^Significant *p*-values (α ≤ 0.05) are bolded. Degrees of freedom are given in square brackets after the *p*-values. Significance was determined through a likelihood-ratio test of null and alternative models, with reference to a χ^2^ distribution.

Abbreviations—***ln*L**, ln Likelihood; ***p***, *p-*value; **N/A**, not applicable.
