# Supplementary material for: Evolutionary pathways to SARS-CoV-2 resistance are opened and closed by epistasis acting on ACE2
Source: PLoS Biol. 2021 Dec 21;19(12):e3001510. doi: 10.1371/journal.pbio.3001510 (PMC8730403; doi:10.1371/journal.pbio.3001510)
Supplement: Attachment — Submitted filename: Castiglione et al_Response to Reviewers.pdf [file pbio.3001510.s017.pdf]

Dear Editors of PLoS Biology,

Thank you for your careful consideration of our manuscript PBIOLOGY-D-21-00553R2 and your decision letter on September 28, 2021. In this newly revised submission, we have performed substantial additional experiments as well as computational analyses, addressing all reviewer concerns. Below we have provided a list of the major additional experiments and revisions, as well as our detailed responses to the reviewer's comments. Please note that all line numbers are in reference to the 'track-changes' version of the manuscript, which was uploaded as a "related" file type. We have also uploaded all source files and supporting information. Please also note that due to the extensive requested revisions, changes were not tracked in the revised abstract.

Overview of major revisions to manuscript

New experimental and computational data: *Figures 2E-G; 3D, 4D, 4E, S3; Tables S3, S4*

New analyses: *Figures 3F, 4F, S2; Table S5*

New references: *#3-5, 48-52, 60-62, 64-65, 70, 72-74, 89-92, 94-95, 105-107*

Major text revisions: *Title, Abstract, description of new results, new caveats in discussion, and more as detailed below.*

Detailed response to Reviewer comments

REVIEWERS' COMMENTS:

Reviewer #1:

In this paper, Castiglione et al. use computational evolutionary and functional experiments to understand how sequence variation in mammalian ACE2 impacts this proteins enzymatic function in addition to its latent capacity to enable SARS-related coronavirus entry. The manuscript is quite nice, I like the figure graphics, and there are some interesting analyses. I really liked the work on epistasis, and also appreciated the conclusion that because of epistasis, simple predictions of what ACE2s a virus may or may not bind are not easy to predict from knowledge within a single ACE2 alone. I do not completely follow some of the logical links between different experiments, and I detail some minor concerns below. Nonetheless, there are some very interesting components in the manuscript that I find compelling and of broad potential interest.

- We thank the reviewer for the constructive criticisms, which have served to strengthen the study. We appreciate the overall positive assessment.

Major points:

1. Title: I don't find the language of "gave rise to" the SARS-CoV-2 pandemic as appropriately describing the findings here. This title suggests a much more proximal role in this ancient genetic variation and the origin of the pandemic. A better title might use language more like "enables susceptibility to SARS-CoV-2 binding"
    - We have revised the title in response to this helpful criticism.

2. Some inaccuracies or statements in describing the state of the field on SARS-CoV-2 evolution that could use correcting or more description:

a. Line 50: RaTG13 is not an "ancestor," but simply another tip on the phylogeny that has been sampled within the same sarbecovirus clade as SARS-CoV-2
   - We have made this correction in the text (lines 46-48)

b. Line 130-131: simply observing that pangolin ACE2 can bind SARS-CoV-2 does not "affirm" any intermediate host status for the direct SARS-CoV-2 line of transmission, which will end up being as much about ecology and contingency as much as anything else. (Though of course, binding/infectivity is a pre-requisitie, observing this binding/infectivity does not mean any individual species is an "intermediate" in the zoonotic sense). There is not yet widespread understanding or evidence that pangolins are direct intermediates in the spillover of SARS-CoV-2 itself, let alone being a result to be "reaffirmed" as in the language here

- These are very important points. We have removed this from the manuscript (lines 140-141)

c. Line 125: there are multiple alleles of R. sinicus ACE2 (e.g. PMID 32699095) that differ in their SARS1 binding capacity - which sequence is being used here should be explicitly stated when introducing the sequence in the main text. And in general, making it clear you tested "a" bat/Rs ACE2 sequence across all language seems important to appropriately represent that there are many different bat ACE2s, including many different alleles even within this one host species

- We have added the accession numbers for all ACE2 sequences at the beginning of the results (lines 133-135), and made clear the species we tested throughout the manuscript (e.g., lines 147, 156)

3. I have a couple important questions about the ACE2 hydrolysis activity assays:

a. Is the ACE2 peptide that is being hydrolyzed 100% conserved across the species being tested? If not, that would raise a major caveat about the assays as currently performed and their physiological relevance

- Great point. Yes, the peptide is 100% conserved. We have added a supplemental figure with the alignment (Fig S2). We have also referred to this new figure in the text (lines 151-152)

b. Is the raw expression of ACE2 among variants within the cell lysates used for activity assays a potential confounder of the measured activities? If so, it may be necessary to quantify and normalize activity by relative expression levels in order to make conclusions about differences in activity of these orthologs.

- We have added a new supplemental figure (Fig. S3) showing that ACE2 orthologs are expressed at highly similar levels within HEK293T cells lysed for the hydrolysis assays. We have mentioned this important control in the results (lines 152-153).

4. Fig. 2F-G, lines 225-227: I do not interpret the difference between the effect of the K353H mutation as measured individually versus on top of the 79/82/83/84 mutations as exhibiting a "more muted effect" in the background of the additional mutations as stated in the manuscript. I think this may be a consequence of me not understanding the proper "scale" that mutations should combine on if additive/non-epistatic. For example, K353H alone has a 50% reduction in the relative RBD association metric - which could also be described as a 2-fold loss of binding relative to WT. When K353H is introduced on top of the 79/82/83/84 mutant background, it has only a 20-30% "raw" decrease in RBD binding (my understanding is this is what the authors are describing as "more muted"), but in fact, this 20-30% decrease in RBD association is >2-fold loss of binding relative to the 79/82/83/84 mutant in both the human and dog ACE2 backgrounds - which would argue the opposite of the authors conclusion, with K353H having a larger relative effect in this combinatorial background. I think in either case, given this assay is not a quantitative binding assay with e.g. thermodynamic measurements, we can't truly know how mutations would additively combine w.r.t. this metric, and so probably making any conclusiosn about potential magnitude epistasis is not fruitful. (This does not impact the sign epistasis in regards to the ACE2 activity assays, as sign epistasis is not subject to this 'uncertain scale of additivity' conern.)

- We agree with these constructive criticisms. We have removed the previous mention of epistasis in viral binding from the results and discussion (lines 283-287; 322-330, 484-487), as this is not necessary to support the central findings of our study. We have edited the manuscript to focus solely on epistasis in ACE2 activity, which as the reviewer points out, is well-established by our experiments.

5. Lines 163-166; 177-179; 182-184 : for the tests of positive selection, are you detecting positive selection within specific branches on the tree, or is it just saying “there is 3ositive selection somewhere on this tree”? Several publications have illustrated positive selection in bats, especially within the Rhinolophus bat reservoirs of sarbecoviruses, which is presumably due to selection on ACE2 sequence specifically to evade sarbecovirus binding and infection. (PMID 32699095, 22438550). In the current manuscript, it is unclear to me whether this positive selection within bats specifically due to viral pressures is giving rise to the positive selection signal, which is then being interpreted across all mammalian orders as evidence for selection related to intrinsic ACE2 function. This should be clarified, and probably the statements implying that this positive selection is specifically due to ACE2 physiological enzymatic activity might need to be tempered. (And these two papers are probably worth discussing specifically in the context of this work including the ACE2 residues they identify as positively selected in these host-virus arms’ races.)

- This is very useful feedback that has strengthened the manuscript. We have run additional analyses without bat ACE2 sequences in the phylogenetic dataset. We still detect significant evidence of positive selection at the same sites (24,79) with similar dN/dS values (w) and posterior probabilities (Table S5). Interestingly, the support for site 79 has gone up after the exclusion of bats. This new analysis is described in the text, along with discussion of the two new papers introduced by the reviewer (lines 185-190, 201-204). We have clarified that this new analysis provides evidence that ACE2 function may be a target of natural selection (lines 209-212)

6. Line 280: it is unclear to me why having a lower native blood pressure would relax selection on ACE2 function. Regardless of the homeostatic ‘set point’, presumably the enzymatic activity of ACE2 is needed anyway to maintain that homeostatic set point. It seems like changes in global blood pressure are probably instead modulated by e.g. the upstream regulation of the peptide itself, or response pathways to the cleaved product, or some other pathway – not the actual catalytic activity of ACE2 itself. And it’s further unclear why a correlation in between body mass and blood pressure establishes any relaxation in constraint – it’s actually almost the opposite, in that it argues that there is some overarching ‘reason’ why smaller animals have lower blood pressure and therefore in fact it is an attuned process, not a relaxation of constraint. This analysis is interesting, but I think the authors might need to more carefully consider how to link it into their overall ‘thesis’.

- We greatly appreciate this thought-provoking constructive criticism. In response, we have revised our interpretations of the data in our manuscript. We agree that low blood pressure is likely an attuned process that would not relax selection on ACE2 function. We have emphasized in the text that high ACE2 function is likely essential for maintaining the low blood pressure of mice and other rodents and discuss how this may impact evolutionary trajectories leading to SARS-CoV-2 immunity (lines 395-404).

The further linkage of all of this to effective population size in lines 369-371 extends this all way too far in my opinion, and the statements on lines 369-371 should just be left out.

- We have removed the statement (now lines 487-490)

7. The setup argued for starting in line 294 does not seem to be 'followed through' with the ASR that was actually performed. Simple measurement of the activity of the reconstructed sequence does not clearly illustrate whether these mutations were differentially tolerated in this ancestor without the loss of activity. It seems this analysis should involve not only reconstructing the ancestor, but also introducing the same mutations as illustrated in Figs. 2F/G into this ancestral sequence to identify whether the 'valley' is absent in this ancestral rodent sequence. I understand that's asking for substantial additional experiment, but it would really increase the interest added by this ASR component to the story - without it, the ASR component doesn't seem to add much additional insight.

- This suggestion has greatly strengthened the manuscript. We have made substantial new experimental additions (Fig 4 D-E) that demonstrates that the rodent ancestor can tolerate all six mouse mutations without any impact to ACE2 activity. This contrasts with human, and mouse, both of which suffer significant decreases in ACE2 activity when reciprocal substitutions are made. We have added a new figure to the text (Fig 4F) that summarizes how these differences impact the evolutionary trajectories of rodents, dog and human ACE2.

Minor points:

1. Line 51: "fusing" with isn't clear what that means,

- we have made this correction to 'recombining' (line 48)

2. Not sure if the use of "pleiotropic" on line 110 is necessarily wrong, but it is sort of tripping me up. Maybe the directionality is reversed? The argument is not that this surface has evolved as a SARS-CoV-2 interaction interface and has pleiotropic consequences for ACE2 activity, but rather sort of the reverse - that evolution of ACE2 sequences due to differences in physiology alters the latent capacity for sarbecovirus receptor usage and susceptibility. Somehow describing this more specifically might enable the authors to avoid using a somewhat contentious word of 'pleiotropy' loosely

- We appreciate this very much. We have used the reviewer's phrasing in the text (lines 116-118)

3. The disconcordance between Fig. 2F/G and Fig. S4 seems important for proper assessment of results. It suggests that the dynamic range of the RBD binding assay is lower than for actual viral entry. It might be worth simply including Fig. S4 within Fig. 2

- We have made this addition to figure 2. Additionally, we have new data that shows that the pseudovirus assay is concordant with flow cytometry data; human mutations confer binding and infection to mouse ACE2 (Fig 2E-F)

Reviewer #2:

In this fascinating article, the authors undertake a series of investigations into the broad host tropism exhibited by SARS-CoV-2, beyond that predicted by studies focused exclusively on comparative sequence analyses of the ACE2 homology to the human ACE2 viral binding interface. In particular, the authors undertake the following major analyses:

1.They investigate ACE2 to SARS-CoV-1 (hereafter SC1) and SARS-CoV-2 (hereafter SC2) RBD binding across a suite of mammalian ACE2 orthologs transfected into 293T cells: human, mouse, bat, dog, pangolin. They show stronger association of SC2-RBD with both human and pangolin ACE2 relative to SC1 and show that the SC1 and SC2 proteins cannot bind mouse not bat ACE2 2.They follow this up in a pseudotype virus system, showing cell entry patterns that recapitulate those described in #1 across these same 5 transfected cell lines.
3.They then measured functional variation in ACE2 enzymatic activity across these same cell lines and found a wide range of hydrolysis activity. Dog, bat, and pangolin displayed low activity compared to human and mouse.
4.They next investigated dN/sS shifts in ACE2 mutational rates across these ACE2 orthologs in a suite of mammals and found evidence of ACE2 under positive selection.
5.They next identified six residues unique to mouse ACE2 and adjacent to the viral RBD to explore the effects of these on virus binding. They took the same approach as in #4 to show that "several" of the sites were under either positive or purifying selection and that primates demonstrated decreased evolutionary rates relative to bats or rodents.
6.They then used site-directed mutagenesis to substitute these six mouse sites in human and dog ACE2. They demonstrate that single mutations had limited effects on SC2 binding and huge deleterious effects on ACE2 hydrolysis activity but when introduced altogether both abolished AC2 binding and rescued the hydrolysis activity of ACE2 due to epistatic activity ('sign epistasis').
7.Now, the authors reasoned that species with higher blood pressure would have a greater need for ACE2 blood pressure regulation and therefore have greater constraints on ACE2 sequence space, so they attempted to correlate ACE2 evolutionary rate with blood pressure. They found that rodents and bats with higher ACE2 evolutionary rates also had lower blood pressure, possibly a mechanism for a less constrained ACE2 fitness landscape in these taxa 8.Finally, in order to explore the hypothesis that rodents may simply have evolved these six mutations by traversing a landscape of permissive mutations, the authors reconstructed ancestral rodent ACE2 and discovered it lacked the six residues of interest (suggesting these mutations evolved recently) but likely had high activity which could have compensated for deleterious mutations on the path to the modern receptor.

The paper is a tour-de-force of intriguing ideas and analyses. There are a few places where I have questions and where the discussion goes a bit too far but on the whole, it is in excellent shape:

-We greatly appreciate the reviewer's encouraging assessment of our study.

1.For #4 above, why were the 107 chosen species selected (or the 89 included in the pruned analyses)? It seems likely that there may be extensive variation in dN/dS across bat species for instance. Some justification for the selected subset is needed.

- We agree, and have added a justification in the results and methods (lines 176-178; 596-598)

2.Following on above, the authors say that "several" of the six residues of interest for mouse ACE2 show evidence of positive or purifying selection across many taxa, with much higher rates in bats and rodents and constraints in humans. Can these findings be summarized in an accessible way, maybe in the supplement?

- This is a helpful suggestion. We have added new details to figure 2C identifying these sites, and have also added figure 2D, which illustrates the higher rates in bats and rodents relative to humans at these sites.

3.Can you make the y-axis on a log scale in Figure 3E? It's unclear whether there is any correlation at all or simply whether rodents and bats just show unusually high evolutionary rates for ACE2 compared with other taxa.

- we have removed this figure from the manuscript, due to comments from other reviewers.

4.The authors talk a lot about bats and mice being "immune" to WT SC2. This is not known for bats. The authors would need to demonstrate lack in infectivity in a live animal model to show this, and in fact, they only show lack of virus entry into a transfected 293T cell line with a single bat species ACE2 ortholog. Since the bat chosen (R. sinicus) is not even the host for the closest known CoV to SC2 (R. affinis), this is a stretch. Additionally, Zhou et al 2020 shows some SC2 virus entry in HeLa cells expressing R. sinicus ACE2. Most of the work in this paper is focused on mouse immunity to SC2 and the relevant residues driving this interaction. I suggest the authors keep most of their speculations limited to mice and not try to extrapolate too far into bats.

- We appreciate this constructive criticism. We have since removed all mention of bats (R. sinicus) being immune to WT SC2.

5.Following on above, I would like to see a 'caveats and limitations' paragraph that mentions how we cannot really determine host range with a limited tool kit in this way. No mention is made of the fact that bats, for instance, might permit SC2 virus entry in some tissues (e.g. GIT tissues) and not others and might have other receptors permitting entry.

- This is an important point. We have added a paragraph to the discussion that discusses this and other caveats (lines 513-529)

6.Additionally, the pangolin fusion hypotheses should be dropped. While pangolin CoV may effectively invade human cells, this paper provides no evidence that it is an intermediate host between bat CoV and WT SC2. It is largely believed that a closer genotype to SC2 than has yet been described is probably circulating in wild bats somewhere still. See MacLeean et al 2021, Boni et al 2020, Andersen et al 2020

- We have removed this interpretation from the results (lines 140-141) and added the hypothesis and supporting references that SC2 is circulating in wild bats (lines 48-49)

7.Finally, the bit about genome engineering of minks or other domestic mammals should be dropped, as this is a big step, of questionable ethics, and moreover, this paper does not demonstrate that it would even work.

- We have removed this from the manuscript (lines 506-512).

Reviewer #3:

This paper seeks to identify amino acid sequence states in the ACE2 protein that confer differences in susceptibility to SARS-Cov2 infection between species and to explain the evolution of these different states in terms of epistasis, natural selection, and pleiotropic effects on ACE2's endogenous enzyme activity.

Understanding the sequence-function relationships underlying ACE2 interactions with SARS-Cov2 is a worthy goal, as is understanding the evolutionary causes of differences in these interactions among species. The subject matter of the paper is therefore of significant potential interest. However, many of the claims are not supported by the experiments

and analyses presented. I'm sorry to say that the claims that have sufficient support after a careful reading are of rather narrow scientific impact and seem best suited for a specialist audience.

- We thank the reviewer for the constructive criticism. In response we have added new experiments and computational analyses and implemented extensive revisions to the text. We believe the additions and clarifications now support the claims.

1. The authors' approach is to transfer 6 amino acid states that exist in mouse ACE2 into human and dog and measure their effects on molecular function. They choose these species and states because: 1) these residues are at sites on the surface of ACE2 that binds the SARS-Cov2 spike protein, 2) human and dog ACE2 have higher relative affinity for SARS-Cov2 than mouse ACE2 does, and 3) mouse is less susceptible to SARS-Cov2 infection than human and dog. With several nice experiments in Figs 1A-E, the authors provide evidence that reinforces premises 2 and 3: mouse ACE2 binds the SARS-Cov2 less efficiently than human and dog ACE2, and cultured cells transfected with mouse ACE2 are less susceptible to pseudovirus infection.

2. But the paper does not show that the six residues are sufficient causes for the difference in affinity and infectivity between the species' ACE2 proteins. The major experiments transfer the 6 mouse states into human and dog ACE2 proteins. However, these "chimeric" ACE2 proteins do not confer cellular resistance to infection nearly as well as the mouse ACE2 protein itself does. Thus, the 6 mouse amino acids can reduce to some extent but not nearly recapitulate the fully resistant cellular phenotype conferred by mouse ACE2 (compare Fig 1E to Fig S4). Further, the reciprocal experiment, where human or dog amino acids at these sites are introduced into the mouse ACE2 was not performed; we therefore do not know the extent to which the 6 states account for the resistance exhibited by the mouse ACE2.

- We thank the reviewer for these comments, which have greatly strengthened the manuscript. We have now performed the reciprocal experiment and find that substituting human variants into these six sites in mouse ACE2 is sufficient to confer binding and infection comparable to WT human (Fig. 2E-F). This provides evidence that these 6 states account for the resistance exhibited by moue ACE2. We describe these results in the text and they impact our interpretation of human and dog ACE2 mutants (lines 230-246; 270-273).

Moreover, the historical substitutions from ancestral states to any derived state is never assayed in any of the species' proteins, as would be required to support the
contention that these substitutions played a causal role in the evolution of increased or reduced affinity/susceptibility.

- We have now performed the suggested experiment, assaying the rodent ancestor containing the derived mouse mutations. Our results show that these mutations played a causal role in the evolution of reduced affinity (Fig. 4D; lines 358-362).

The evidence therefore establishes that a small number of residues in mouse ACE2 can partially reduce affinity and infectivity when introduced into human or dog. But it does not support a causally sufficient role for the mouse states in resistance by the mouse ACE2. This observation is interesting in terms of ACE2 sequence-function relationships, but it does not have clearly interpretable implications for genetic/biochemical causality that underlies differences between species or the evolution of those differences.

- As described above, we have demonstrated that these sites are causally sufficient for the resistance of mouse ACE2.

3. A central claim of the paper is that the 6 states interact epistatically in producing the reduced affinity of ACE2 for SARS-Cov2 and the reduced enzyme activity. These claimed epistatic interactions are then said to explain why susceptible species have not evolved genotypes resistant to SARS-Cov2. The evidence for epistasis with respect to affinity is not convincing, because detecting epistasis requires a significant deviation from a well-founded expectation for a quantitative phenotype that would be observed in the absence of epistasis. For example, in the absence of

epistasis, one would expect the effect of a combination of mutations on the free energy of binding to be the sum of the energetic effects of each mutation introduced singly; the effect on Kd is expected to be multiplicative. But no such expectation or test is provided here to show that the effects of combinations are different from the effects that would arise if there were no epistasis. For binding, the assay is a complex one that does not directly quantify affinity, occupancy of the bound state, etc. reasons. The paper claims an epistatic interaction for binding between mutation at site 353 and those at the other sites, but there is no apparent epistasis at all on this case: site 353 and the set of the other mutations each reduce affinity, and they reduce affinity to a greater extent when combined, precisely as expected with no epistasis. The data do show that 5 of the 6 mouse states produce no clear effect on binding when introduced singly, and they do reduce binding when combined with each other or with the sixth state which affects affinity on its own. This does not necessarily indicate epistasis. Suppose the assay has an intrinsically nonlinear dose-response relationship (such as a hyperbolic or sigmoidal relationship, as is expected in any saturable assay); in such a case, single mutations that each have a moderate effect on affinity may produce no detectable reduction in the signal of binding, if introduced singly into a high-affinity protein, but when introduced into a protein whose affinity has already been weakened by other mutations, that effect will become apparent. Further, there is no evidence for epistasis in the infectivity assay shown in Fig S4, where progressively including more mutations progressively reduces infectivity. These observations do not rule out the possibility of some kind of relatively subtle epistasis with respect to the magnitude of mutations' effects, but they do not establish it. The paper therefore provides no persuasive evidence of epistasis for the phenotype of ACE2 affinity for SARS-Cov2 or susceptibility.

In the absence of quantitative knowledge concerning the expected phenotype when nonepistatic mutations are combined, one could still provide some evidence of epistasis if the sign of the effect of a mutation differs when introduced into different genetic backgrounds; the only way sign epistasis can arise without epistasis is for the underlying relationship between the measured phenotype and underlying biochemical effects to be nonmonotonic, and that is unlikely in the case of apparent binding in these assays. The authors do observe sign epistasis for the catalytic phenotype, because the mouse state at site 353 reduces activity on its own when introduced into human ACE2 but increases it when introduced into the context of four other mouse states. Thus, the authors should make no claim for epistasis with respect to binding or infectivity, but they can make a limited claim for epistasis of this mutational combination with respect to catalysis.

- We agree, and greatly appreciate the reviewer's comments. We have removed the previous mention of epistasis in viral binding from the results and discussion (lines 283-287; 322-330, 484-487), as this is not necessary to support the central findings of our study. We have edited the manuscript to focus solely on epistasis in ACE2 catalysis, which as the reviewer points out, is well-established by our experiments.

4. The paper's central evolutionary narrative is that the lack of evolved resistance in humans and dog is attributable to a claimed pleiotropic cost incurred by reducing ACE2's affinity for SARS-Cov2. Mice are claimed to be free of this pleiotropic constraint, because the function of ACE2 in their cardiovascular system is different from that in humans and many other mammals. The data do not coherently support this premise, for several reasons.

a. Fig. 1 shows that the mouse ACE2 actually has higher peptidase activity than human and dog, not lower, as would be required for the peptidase-versus-affinity tradeoff to explain the evolution of viral resistance in mouse but not in humans.

reasons.

- We greatly appreciate these constructive criticisms. We have revisited our interpretations of ACE2 activity in mice. We propose that high ACE2 activity may have evolved in rodents to facilitate Ang-II degradation at a higher level than other species. Since Ang-II induces hypertension in mice, high ACE2 activity may therefore help maintain the lower blood pressure setpoint in mice relative to other species (lines 395-404).

b. The authors observe reduced ACE2 enzyme activity when the 6 mouse states are introduced into the human ACE2, but no such reduction is observed in dog. This means that there is no intrinsic association between the two phenotypes, as is required to claim that humans and dogs have not evolved resistance to SARS-Cov2 because of antagonistic pleiotropy related to ACE2 activity.

- We truly value this feedback, as it raises an interesting observation that dogs, but not humans, could potentially diminish their susceptibility to SARS-CoV-2 infection. This has allowed us to reframe the emphasis of our narrative by comparing the evolutionary trajectories of human and dog ACE2. We have

done so by adding new figures (Fig. 3F, Fig. 4F) completely revising our abstract, the results (lines 318-322, 345-347) and discussion (lines 458-460)

c. The observation that the 6 mouse states decrease SARS-Cov2 affinity and reduce peptidase activity in human ACE2 would at best imply only that humans may be unlikely to evolve reduced affinity by acquiring these particular six mouse states. This does not establish that they could not do so via other mutations that may not affect peptidase activity. reasons.

- We certainly agree that this is a possibility. We have added a qualifying statement to the text that other combinations may exist which avoid this trade-off (lines 422-423, 524-526).

d. The fact that the six mouse states do not have the deleterious effects on dog ACE2 indicates that the states at other sites in the protein can prevent the deleterious effect of the six mouse states, indicating that human ACE2 might be able to acquire the 6 mouse states if it also acquired other residues that have a similar modifying or buffering effect. Thus, the authors' data establish only that ***human*** ACE2 could not reduce its SARS-Cov2 affinity without incurring pleiotropic effects on affinity by acquiring only the 6 mouse sites. A general statement about acquisition of resistance per se therefore cannot be justified.

- We agree with the interpretation that humans could have potentially evolved the 6 mouse states if they also evolved permissive mutation(s) that buffer the negative effects. As stated in our responses above, we have added a qualifying statement to the text that other combinations may exist which avoid this trade-off. We have also reframed the emphasis of our narrative by comparing the evolutionary trajectories of human and dog ACE2 (Fig. 3F, Fig. 4F; (lines 37-39, 318-322, 345-347, 458-460)

-

5. The authors claim that evolution of the ACE2 protein and several of the six states in particular has been driven by positive selection. They base this claim on the results of two kinds of model-based likelihood ratio test, the branch-sites test and the sites test. However, both of these tests have been shown in the literature to be unreliable, with very high propensities to return false positive conclusions under realistic conditions. These methods therefore do not provide reliable evidence for the claims about selection. It is true that these methods have been widely used in the past as evidence for positive selection; given the recent findings, however, they should no longer be used. See Witosky et al, Synonymous site-to-site substitution rate variation dramatically inflates false positive rates of selection analyses: ignore at your own peril, MBE 2020; Venkat et al, Multinucleotide mutations cause false inferences of lineage-specific positive selection, Nature
Evol Evol 2018.

- These are important concerns. To address the effect of synonymous rate variation, we have run the additional analyses recommended by Witosky et al.. This new analysis demonstrates that synonymous variation does not confound our results of positive selection (lines 181-184). We have now explicitly acknowledged in the text that the existence of multinucleotide substitutions could have disrupted our ability to detect bona fide positive selection, and state that this necessitates our functional characterization experiments (lines 233-235)

-

6. The authors claim that the 6 mouse states could have been selectively accessible in rodents because rodents have lower systolic blood pressure than other mammals, which could result in lower selective pressure to maintain ACE2 function, thereby reducing the deleterious costs of the 6 states. The authors provide as evidence of this relaxed constraint hypothesis a higher ratio of nonsynonymous to synonymous rates of evolution at these sites in rodents and bats compared to other mammals. In addition to concerns about these tests as discussed above, the analysis appears to have been performed only on the subset of sites that differ in amino acid state between mouse and other mammals, which are tautologically expected to have higher rates of nonsynonymous substitution in rodents.

- We agree that this is an obvious result. We have since removed this analysis from the paper (lines 393-395). We have however, conducted a similar analysis in the new figure 2D, simply to quantitatively illustrate the lower rates of nonsynonymous substitution in primates at these sites specifically. We understand that this result is expected given the lack of variation in primates, but we maintain that the computational quantification is necessary to demonstrate this expectation.

A second problem is that the authors also state that unlike other mammals, mice do not exhibit a vasodilatory response to Ang-(1-7), the product of ACE2 hydrolysis. This observation seems to contradict the low blood pressure hypothesis for putative relaxed selective constraint: if ACE2 does not mediate vasodilation, then it is unclear why low blood pressure should produce any relaxed constraint at all.

- We greatly appreciate these constructive criticisms. We have now clarified in the text that mouse ACE2 plays an important role in the degradation of Ang-II, which promotes vasoconstriction and hypertension in mice. Accordingly, we have revised our interpretation of relaxed selection in mouse ACE2. We propose that high ACE2 activity may have evolved in rodents to facilitate Ang-II degradation at a higher level than other species. Since Ang-II induces hypertension in mice, high ACE2 activity may therefore help maintain the lower blood pressure setpoint in mice relative to other species (lines 395-404).

7. Based on the observation that the 6 mouse states that reduce the peptidase activity of human ACE2 are not located at the protein's catalytic active site, the authors state that this effect must be mediated by indirect structural effects. However, the most plausible mechanism by which mutations would have this effect would be by impairing substrate binding, which takes place at the portion of the ACE2 surface where SARS-Cov2 binding occurs. This would not reflect a surprising mechanism, and it would not be indirect, as it would involve mutations at the protein's surface directly compromising interactions with the substrate at that surface.

- This is an important hypothesis. We have removed this previous language (lines 423-424) and have added a discussion on this topic to the manuscript (lines 411-417).

8. The authors state that it is surprising that the 6 mouse residues reduce SARS-Cov2 affinity when introduced into either human or dog, because human and dog have different states at most of these sites. They say that this indicates that homology-based reasoning is a poor predictor of proteins' affinity. But the observation that the human and dog states are different is not at all surprising - all it means is that there are multiple amino acid states per residue that are compatible will affinity higher than that conferred by the mouse states. It is very common in multiple sequence alignments to observe some sequence variability at functionally important sites in a protein, such as exchanges between hydrophobic states in a protein's core, or between polar states on a protein's surface, or between donor states (or acceptors) in hydrogen-bonding residues. No selective or epistatic explanation is required to account for this variation - some sequence degeneracy of the functional property is all that is required.
The authors' results do show that a search for strict conservation of a single state between proteins with similar affinity is not a reliable guide to identifying sequence sites that contribute to that phenotype, but it would be very naïve to think that it would be. A further consideration related to sequence variability at these sites is that affinity for SARS-Cov2 could not possibly have been a source of long-term constraints that affect sequence variation among species, because the virus did not emerge until 2019. There is no reason that we should expect sites that contribute to affinity to be strictly conserved over evolutionary time.

- We greatly value this constructive criticism from the reviewer. We have made revisions throughout the manuscript emphasizing this point (e.g., lines 23-26, 72-79), which is very important as it supports the central revised narrative of our manuscript— despite this degeneracy, a rare combination exists in mouse ACE2 capable of conferring immunity. As the reviewer points out, there is no reason to believe that this combination would be off limits to other species a priori. Thus, what are the constraints that limited this sequence to rodents alone? Thanks to the reviewers' comments, our expanded study now focuses on the evidence that antagonistic pleiotropy in human ACE2 limited this combination from evolving.

9. The authors refer to a "functional convergence" between dog and human in their shared susceptibility to SARS-Cov2. But the paper suggests that susceptibility and high ACE2 affinity is ancestral, with a reduction in these phenotypes in the lineage leading to mouse. Susceptibility is therefore not convergent but a retained ancestral state.

- We agree with this point. Our new results provide further evidence of this ancestral susceptibility as described in the text (lines 358-362)
