# Supplementary material for: Evolutionary pathways to SARS-CoV-2 resistance are opened and closed by epistasis acting on ACE2
Source: PLoS Biol. 2021 Dec 21;19(12):e3001510. doi: 10.1371/journal.pbio.3001510 (PMC8730403; doi:10.1371/journal.pbio.3001510)
Supplement: Attachment — Submitted filename: Response to Reviewers.pdf [file pbio.3001510.s018.pdf]

REVIEWER'S COMMENTS:

Reviewer #1:

The authors have performed considerable work to streamline the manuscript in light of reviewer comments. I still have some various nitpicky details, of minor to modest importance, as I still feel there are some errant claims and comments in the manuscript. In addition to the specific calls below, I feel as a general tonal comment, speculations made beyond conclusions across the manuscript stray a bit far and could be reined in for a tighter interpretation. However, I am better able to appreciate the logical flow of this manuscript than in its prior form. My additional comments are below - all should be achievable via changes in writing with no further experiments requested from my reading. Comments are in order of reading, not importance.

1. Most dramatically in the title and abstract, but also used throughout the manuscript: I dislike the use of "immunity" to describe resistance to SARS-CoV-2 infection in ACE2 variants that resist binding by SARS-CoV-2. In the virology and immunology field, the suggestion of "immunity" would immediately invoke something about innate or adaptive immune system conferring "immunity" to infection by the virus. I believe instead of "immunity", phrasing about "susceptibility' versus perhaps "resistance" (though there may be a better word still than resistance) would be much preferred over "immunity"

We agree with this criticism and have made the changes throughout the manuscript (e.g., lines 1-2, 37, 282).

2. Lines 43-46: RaTG13 is from R. affinis, not R. sinicus. There is no supported role for recombination with a pangolin virus nor support for pangolin as an intermediate source at this time. To establish zoonotic origin, should be fine to just leave it as referencing circulation of related viruses in several Rhinolophus bat species (at this point, can cite other sarbecoviruses more closely related than RaTG13 as well, and probably don't need to name specific isolates as much as generally point out closely related bat viruses). If needing to discuss why pangolin is included, I would summarize pangolins at this point as another species susceptible to SARS-CoV-2-related CoV infection and spillover, though the routes of exposure and transmission in pangolin communities (e.g. natural circulation, animal trafficking, etc.) are at present unclear.

We greatly appreciate the reviewer’s clarifications on this subject, and we have made the corresponding changes (lines 45-49).

3. Line 64-67: interaction interface of SARS1 and SARS2 is homologous, and this dichotomy between SARS1 susceptibility to a single mutation and SARS2 targeting multiple hotspots is not a supported claim to my knowledge. SARS1 structure papers also discuss "hotspot" ACE2 residues for interaction, and citations 24-26 don't establish that the single mutation in citation 23 that reduces SARS1 binding does not also reduce SARS2 binding. This differentiation between SARS1 and SARS2 structural mechanisms of ACE2 binding is not well supported and is not necessary to the paper.

We greatly appreciate the reviewer’s clarifications on this subject, and we have removed this from the text changes (lines 67-68).

4. Line 72-74: somehow missing in this section is consideration that for most species, apart from natural bat reservoirs, there might not be selective pressure to evade binding by SARS-related coronaviruses because these viruses do not naturally circulate in these other species. The current description around antagonistic pleiotropy seems to assume that there naturally would be selective pressure in all of these species to evade binding by SARS-related coronaviruses, thereby requiring some additional explanation, but there need not be any reason in species beyond bats why resistance has evolved that is proximally related to coronavirus susceptibility

This is an important point. We have made the corresponding changes and edited this introductory paragraph in this light (lines 74-82).

5. Line 126 and on: Shortening of SARS-CoV-2 and -1 to simply "CoV-2" is not standard. Ok in the figure legends for conciseness, but I would avoid it in the written text.

This has been corrected (lines 133-134)

6. Fig. 1: the first time pangolin's and what I have to assume is a bat's silhouettes appear, there is no written label to clarify what ACE2 is being shown. Silhouettes are not sufficient

This has been corrected (Figure 1D).

7. Line 238-240: result seems overstated - K353H does still have a noticeable effect on RBD association in the flow-based assay. Given likely nonlinear relationship between true RBD:ACE2 affinity and labeling in this flow-based assay, this difference in complete versus partial abolishment by K353H could be more about starting affinity of SARS-CoV-1 versus SARS-CoV-2 more than an underlying difference of sensitivities of these viral RBDs to this mutation. True affinity measurements would be needed to support this claim.

This claim has been removed from the text (line 257-260)

8. What error bars represent, the number of replicates, and whether they represent technical versus biological replicates should be clarified in all figure legends

This has been done.

9. The orange circle in the Fig. 4B cartoon is indicative of the primate-rodent ancestor (lateral movement is irrelevant on the tree, so it is equivalent in position to the node of the human/mouse common ancestor). Assuming the black wedge is assumed to represent proliferation of rodents, the orange circle should be moved up to the apex of this black wedge.

This has been done.

10. In the paragraph starting 357 - this hypothesis doesn't "explain" why these six substitutions occurred, because the data suggests that although they maintain the higher catalytic rate, they don't

further improve it - is that correct? If so, this paragraph currently is written as though explaining why these mutations occurred in the mouse lineage but not the dog - but in either case, as shown in Fig. 4F, in both backgrounds the six mutations have no net change on the base activity - so this is insufficient to explain their occurrence in mouse but not in dog which in my current reading is the 'purpose' of this paragraph

This is a very helpful critique. We have revised this paragraph to clearly state that these mutations likely did not evolve as an improvement to drive higher ACE2 activity; high activity already existed due to blood pressure constraints, and this may have been a permissive phenotype absent from non-rodent species (lines 402-409). In that revised text, we have also directed the reader to the pre-existing elaboration of the support for this theory in the discussion (lines 462-476)

11. In places such as line 421, the assumption is that the mouse combination is the only route to resistance to SARS2 binding. This is assuredly not the case, as bat ACE2 alleles that abolish SARS-CoV-2 binding use different ACE2 substitutions. Therefore, words such as "required" are incorrect (implying necessity), but rather language here and elsewhere should indicate the mouse solution is one "sufficient" way to restrict binding. I now see this is caveated as a Discussion point, but that doesn't offset the need to use appropriately cautious language when discussing the results.

We have now addressed this important point here (line 452) and elsewhere (e.g., lines 281, 440)
